# A scoping review of suicide prevention interventions for Latinx adults: strategies, gaps, and future directions

**DOI:** 10.3389/fpubh.2025.1481904

**Published:** 2025-02-19

**Authors:** Airín D. Martínez, Samantha Kloft, Pablo Fernandez, Parastoo Dezyani, Alandra Ricci, Delvis J. Hernández, Kelley Cunningham

**Affiliations:** ^1^Department of Health Promotion & Policy, School of Public Health & Health Sciences, University of Massachusetts, Amherst, MA, United States; ^2^Institute for Sexual and Gender Minority Health and Wellbeing, Feinberg School of Medicine, Northwestern University, Chicago, IL, United States; ^3^Division of Violence and Injury Prevention, Massachusetts Department of Public Health, Boston, MA, United States

**Keywords:** adults, Hispanics, interventions, Latina, Latino, Latinx, mental health, suicide prevention

## Abstract

Suicide rates among Hispanic/Latina/o/x (Latinx) individuals in the United States have escalated significantly, becoming the fifth leading cause of death by 2020. This trend underscores the necessity for culturally responsive suicide prevention (SP) interventions tailored to Latinx persons’ unique sociodemographic characteristics. We synthesized the current literature on suicide prevention (SP) interventions for U.S. Latinx adults (18+ years). Our objective is to identify strategies, culturally sensitive approaches, and interventions that mitigate suicidal ideation, attempts, and deaths among Latinx adults. Following PRISMA guidelines, we conducted a comprehensive search across six electronic databases (CINAHL Complete, PubMed, PsycINFO, SocAbstracts, Sociology Database, and Web of Science), focusing on peer-reviewed articles published between 2000 and 2024 that described or evaluated SP approaches for Latinx adults (ages 18–64) in the United States. The review was structured according to the 2022 CDC Suicide Prevention Resources for Action, Strategies and Approaches to Suicide Prevention. Our search produced 4,739 articles, of which 155 were included for full-text review. During full-text review, 34 articles were included for the final sample. We randomly selected 10 articles and coded them to check for inter-rater reliability (*r* = 0.90). Analysis revealed that most SP interventions for U.S. Latinx adults align with the CDC strategy to “Identify and Support People at Risk.” The majority targeted late adolescents and early adults at the individual level. The predominant cultural adaptation was the translation of existing SP interventions into Spanish. These findings highlight the pressing need for more culturally responsive Latinx SP interventions that address other CDC strategies at the community and structural levels. Future research and intervention development should focus on creating comprehensive, culturally nuanced approaches that extend beyond individual-level interventions and language translation to address the complex factors contributing to Latinx adults’ suicide risk.

## Introduction

The Hispanic/Latina/o/x (from now, the gender inclusive term, *Latinx*) population is a multiracial, multilingual, and multinational racialized ethnic group in the United States (1). There are about 60.6 million Latinx persons in the United States (2), and they have been the second largest racial-ethnic group in the United States since 2003 (3). In California and Texas, Latinx people make up >45% of the population (2). Four out of five Latinx persons are U.S. citizens, while the number of immigrants from Latin America has declined since 2007. The majority of Latinx persons in the U.S. are Mexican-origin (61.9%), followed by Puerto Ricans (9.7%) and Central Americans (9.5%) (4).

There has been an increase in suicidal ideation, suicide attempts, and deaths by suicide among historically marginalized populations in the United States, particularly Latinx adults. Latinx women and youth are reporting more suicidal ideation and women are attempting more suicides, while Latinx working-aged men have had a steady increase in death by suicide for the last 10 years (5). Explanations for this increase include cultural stressors, economic instability, mental health stigma, social isolation, and less access to behavioral healthcare services (6, 7). Public health suicide prevention (SP) interventions seek to reduce the presence of individual risk factors, teach emotion regulation to very young children (ages 5–7), train the public to identify signs of suicide, and refer people in crisis to services.

This public health issue requires an investigation of available SP interventions, and specifically, those interventions that have targeted the Latinx English- and Spanish-speaking population. We conducted a scoping review of evidence-based strategies, culturally responsive approaches, interventions, and programs that mitigate suicidal ideation, attempts, and deaths in U.S. Latinx adults. *Our main research question is: What is the availability of evidence-based suicide prevention (SP) strategies, culturally responsive approaches, interventions, and programs for English- and Spanish-speaking Latinx adults in the United States?* We focus on adults (18+ years of age) because prior reviews written about SP approaches and interventions for the U.S. Latinx population focused on Latinx youth (8, 9). To our knowledge, there is yet to be a review about adults, which is unfortunate since the highest deaths by suicide in the Latinx population is among working-aged men. We include suicide prevention (SP) evidence-based strategies, culturally responsive approaches and interventions and programs at the structural, group and interpersonal levels that seek to reduce suicidal ideation, suicide attempts and deaths in English- and Spanish-speaking Latinx adults in the United States. We include strategies, approaches, interventions and programs that were *created for* and/or *included* the U.S. Latinx population between 2008 and 2024. We evaluate the availability of such interventions, whether SP interventions are created from rigorous research, and their efficacy in reducing suicidal ideation, attempts, and deaths by suicide. We end by proposing future research and interventions specifically tailored to address the unique needs of U.S. Latinx populations.

## Methods

Following the PRISMA guidelines, we systematically conducted a comprehensive search across six electronic databases: CINAHL Complete, PubMed, PsycINFO, SocAbstracts, Sociology Database, and Web of Science, to identify studies pertinent to SP interventions including or exclusively created for Latinx adults. Our search terms were: suicide AND (hispanic OR latin*) AND (approach OR intervention OR prevention OR “cultural* adapt*”). We consulted a university librarian to help us modify search terms and select the appropriate databases. The inclusion criteria were: (1) peer-reviewed journal articles published between 2000 and 2024, (2) that described or evaluated SP approaches, interventions, evidence-based practices, or cultural adaptations, (3) for Hispanic and/or Latina/o/x adults, or a specific Latinx subgroup (e.g., Mexican Americans, Puerto Ricans, etc.); (4) ages ≥ 18, and (5) in the United States.

**Figure 1:**
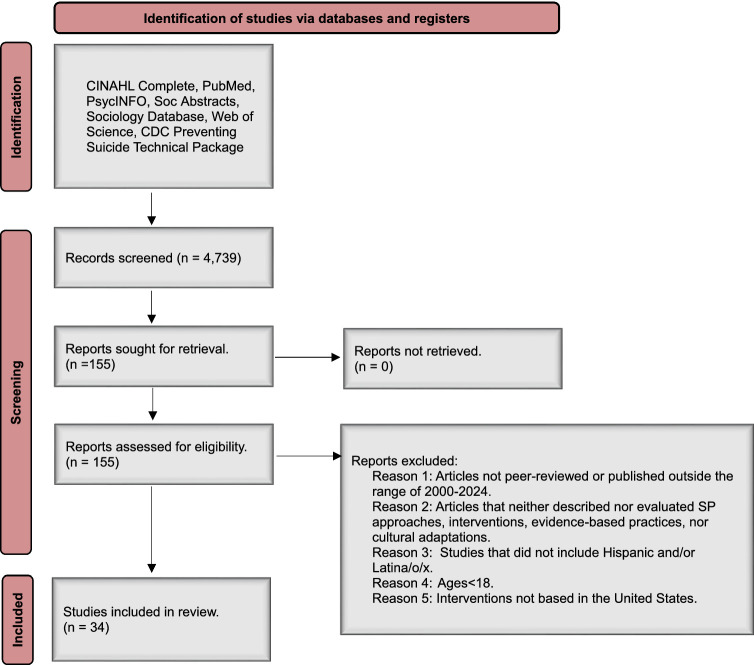
PRISMA flow diagram of scoping review of Latinx suicide prevention approaches, cultural adaptations and interventions in the United States.

Data extraction was performed to ensure adherence to the PRISMA methodology. Online articles were searched manually without the aid of specific software. Microsoft Excel was utilized to organize and manage the data for all collected articles. Four authors independently (ADM, SK, PD, DH) assessed each selection criteria using a rubric with the inclusion criteria built into a Qualtrics survey. In Microsoft Excel, a color-coded system was employed to categorize articles based on Qualtrics survey results. Articles that did not meet the inclusion criteria were marked in red, those that met all inclusion criteria were coded in green, and articles requiring further review by the research team were highlighted in yellow. We included both concept papers that described suicide prevention diagnostic and intervention approaches and empirical papers that evaluated or tested suicide prevention strategies and interventions. We did not exclusively focus on a specific study design as we anticipated there not being many culturally specific or culturally modified suicide prevention approaches, strategies and interventions for the U.S. Latinx adult population. Additionally, we excluded studies that focused exclusively on adolescents or non-U.S. populations, as well as gray literature, including non-peer-reviewed articles, dissertations, and conference abstracts. This decision reflects our focus on evidence-based practices specific to U.S. Latinx adults. We then synthesized and analyzed the existing literature (see Figure 1).

Our search produced 4,739 articles. Our first step was to have a team of co-authors (PF, ADM, SK, AR) screen the 4,739 titles, abstracts, and keywords. We randomly selected 10 articles and coded them to check for inter-rater reliability (*r* = 0.90). Of the 4,739 articles, 155 were included for full-text review based on relevance to our research question and the inclusion criteria. We included 12 articles that discussed SP approaches, strategies or interventions for Latinx youth in secondary school if the sample included young adults (18–22 years). Of the 155 articles, 39 matched the five criteria to be included in the scoping review. We excluded five additional articles because suicidal behavior in Latinx adults was not the primary outcome, or the article did not discuss an approach, intervention or cultural modification to prevent suicide in Latinx adults.

## Results

We evaluated each individual article by identifying how each approach, intervention or cultural modification addressed one or more of the seven 2022 CDC Suicide Prevention Resources for Action Strategies and Approaches (10) (see Table 1). We conducted an additional search of the CDC Suicide Prevention Resources for Action to identify evaluated strategies and approaches involving a Hispanic/Latina/o/x subsample. This yielded no additional articles for our review that we did not already have in our sample. Thirty-four (11) articles were included in the final review.

**Table 1:** CDC suicide prevention strategies implemented in US Latinx interventions, approaches and modifications*.

	CDC strategy to achieve and sustain substantial reductions in suicide across 34 studies						
	Strengthen economic supports	Create protective environments	Lessen harms and prevent future risk	Promote healthy connections	Teach coping and problem-solving skills	Improve access and delivery of suicide care	Identify and support people at risk
Number of interventions	0	3	4	9	11	14	25

*19/34 (55.9%) interventions include multiple CDC suicide prevention strategies to achieve and sustain substantial reductions in suicide; 15/34 (44.1%) interventions focus on a single CDC suicide prevention strategy.

### Organization of Review

The following sections discuss the articles based on specific populations that provide SP care and support to Latinx adults (e.g., clinicians, clergy) and SP approaches, cultural modifications, strategies and interventions that address specific Latinx adults (e.g., late adolescents/young adults, veterans, families, etc.). We end each section by identifying which CDC SP Approach that each article addressed. Table 2 provides a summary of each article. We chose to display our data extraction information in this table format specifically to highlight how each study aligns with the 2022 CDC Suicide Prevention Resources for Action, and more specifically the strategies and approaches to achieve and sustain substantial reductions in suicide.

**Table 2:** Methodological, sample, and CDC strategy characteristics for suicide prevention programs and interventions targeting Latinx adults (2000–2024).

Authors (Year)	Latinx study participants # (%)	Participant age	CDC strategy(ies) to achieve and sustain substantial reductions in suicide	CDC approach(es) to achieve and sustain substantial reductions in suicide	Latinx-specific or includes Latinx	Sample size	Study design	Improve suicide behavior (Y/N)
Clinician-based interventions								
Almeida et al. (15)	2 (9.1%)	24–38	Identify and support people at risk	Train gatekeepers	Includes Latinx	22	Quantitative	Y
Matthieu et al. (16)	7 (10.4%)	24–74	Identify and support people at risk Improve access and delivery of suicide care	Train gatekeepers Plan for safety and follow-up after an attempt Create safer suicide care through systems change	Includes Latinx	70	Mixed methods; qualitative, semi-structured interview and a quantitative, self-report survey	Y
Reyes-Portillo et al. (17)	Unspecified	30–39	Identify and support people at risk	Plan for safety and follow-up after an attempt	Includes Latinx	40	Quantitative	Y
Waitzkin et al. (18)	118 (98.3%)	30–59	Identify and support people at risk Improve Access and delivery of suicide care	Plan for safety and follow-up after an attempt Create safer suicide care through systems change	Includes Latinx	120	Mixed methods; survey & ethnography	Unspecified
Late adolescent-based interventions								
Aseltine & DeMartino (21)	Unspecified	13–21	Identify and support people at risk Teach coping and problem-solving skills	Train gatekeepers Support social–emotional learning programs	Includes Latinx	2,100	Quantitative; quasi-experimental design	Y
Etter et al. (27)	305 (14.3%)	12–20	Identify and Support People at Risk	Provide therapeutic approaches	Includes Latinx	2,134	Quantitative; prospective cohort study	Y
Ford-Paz et al. (19)	18 (100%)	14–18; 27–61	Identify and support people at risk Improve access and delivery of suicide care Promote Healthy Connections	Train gatekeepers Create safer suicide care through systems change Engage community members in shared activities	Latinx-Specific	18	Qualitative; community-based participatory research	Y
Humensky et al. (24)	55 (100%)	12–18	Teach coping and problem-solving skills Promote healthy connections Lessen harms and prevent future risk	Teach parenting skills to improve family relationships Engage community members in shared activities Report and message about suicide safely	Latinx-Specific	55	Mixed methods; quantitative assessments, qualitative focus groups and semi-structured interviews	Y
Humensky et al. (23)	107 (100%)	11–18	Identify and support people at risk Teach coping and problem-solving skills	Plan for safety and follow-up after an attempt Teach parenting skills to improve family relationships	Latinx-Specific	107	Mixed methods; community-based uncontrolled trial	Y
McCauley et al. (22)	48 (27.5%)	12–18	Identify and support people at risk	Plan for safety and follow-up after an attempt	Includes Latinx	173	Quantitative; randomized clinical trial	Y
Sekhar et al. (26)	2,684 (20.8%)	13–21	Identify and support people at risk	Plan for safety and follow-up after an attempt	Includes Latinx	12,909	Quantitative; randomized clinical trial	Y
Sise et al. (28)	31 (4.3%)	18+	Promote healthy connections Lessen harms and prevent future risk	Promote healthy peer norms Report and message about suicide safely	Includes Latinx	708	Quantitative; cross-sectional survey	Y
Young adult-based interventions								
Chesin et al. (32)	312 (38%)	18–25	Lessen harms and prevent future risk	Report and message about suicide safely	Includes Latinx	818	Quantitative; cross-sectional survey	Unspecified
De Luca et al. (31)	Unspecified	18–35	Improve access and delivery of suicide care	Provide rapid and remote access to help	Includes Latinx	26,292	Quantitative; cross-sectional survey	Unspecified
Frick, Butler & deBoer (29)	218 (13.5%)	18–22	Identify and support people at risk Improve access and delivery of suicide care Promote healthy connections Create protective environments	Train gatekeepers Respond to crises Create safer suicide care through systems change Engage community members in shared activities Create healthy organizational policies and culture	Includes Latinx	1,607	Quantitative; pilot study employing pre/post outcomes evaluation	Y
Rivero et al. (30)	8 (7%)	18–22	Identify and support people at risk Teach coping and problem-solving skills Promote healthy connections Lessen harms and prevent future risk Create protective environments	Respond to crises Plan for safety and follow-up after an attempt Support social–emotional learning programs Teach parenting skills to improve family relationships Promote healthy peer norms Intervene after a suicide (postvention) Report and message about suicide safely Create healthy organizational policies and culture	Includes Latinx	108	Quantitative; case study	Y
Silva et al. (20)	56 (100%)	18+	Identify and support people at risk	Provide therapeutic approaches	Latinx-Specific	56	Quantitative; cross-sectional validation study	N
Vargas et al. (33)	1,607 (100%)	18+	Improve access and delivery of suicide care	Create safer suicide care through systems change	Latinx-Specific	1,607	Qualitative; community-engaged	Y
Adult-based interventions								
Arias et al. (38)	114 (13%)	18+	Identify and support people at risk Improve access and delivery of suicide care	Respond to crises Provide rapid and remote access to help	Includes Latinx	874	Quantitative; quasi-experimental clinical trial	Unspecified
Costantino et al. (36)	272 (100%)	65–94	Improve access and delivery of suicide care	Create safer suicide care through systems change	Latinx-Specific	272	Quantitative; observational study	Y
Dueweke et al. (39)	78 (100%)	21–70	Teach coping and problem-solving skills	Support social–emotional learning programs	Latinx-Specific	78	Quantitative; randomized controlled trial	Unspecified
Goodsmith et al. (37)	407 (40%)	18+	Identify and support people at risk Improve access and delivery of suicide care Promote healthy connections	Respond to crises Increase provider availability in underserved areas Provide rapid and remote access to help Engage community members in shared activities	Includes Latinx	1,018	Mixed methods; longitudinal observational study	Unspecified
Gwaltney et al. (34)	4 (5%)	18+	Identify and support people at risk Improve access and delivery of suicide care	Respond to crises Provide rapid and remote access to help	Includes Latinx	86	Quantitative; randomized crossover-equivalence study	Unspecified
Kimbrel et al. (11)	6,408 (18%)	18+	Identify and support people at risk Improve access and delivery of suicide care	Respond to crises Provide rapid and remote access to help	Includes Latinx	35,654	Quantitative; prospective cohort study	Unspecified
McCourt et al. (40)	Unspecified	All Ages	Create Protective Environments	Reduce access to lethal means among persons at risk for suicide	Includes Latinx	Unspecified	Quantitative; observational study	Y
Silva et al. (35)	16 (100%)	24–63	Identify and support people at risk Improve access and delivery of suicide care	Respond to crises Provide rapid and remote access to help	Latinx-Specific	16	Mixed methods; prospective observational study	Unspecified
Family-based interventions								
Diamond et al. (41)	20 (15.5%)	12–18	Identify and support people at risk Teach coping and problem-solving skills	Provide therapeutic approaches Support social–emotional learning programs Teach parenting skills to improve family relationships	Includes Latinx	129	Quantitative; randomized controlled trial	Y
Garcia et al. (42)	Unspecified	Unspecified	Identify and support people at risk Improve access and delivery of suicide care Teach coping and problem-solving skills Promote healthy connections	Provide therapeutic approaches Create safer suicide care through systems change Teach parenting skills to improve family relationships Engage community members in shared activities	Includes Latinx	Unspecified	Qualitative; community-based participatory research approach	Unspecified
Vidot et al. (43)	746 (100%)	13–14	Teach coping and problem-solving skills	Support social–emotional learning programs Teach parenting skills to improve family relationships	Latinx-Specific	746	Quantitative; randomized control trial	Y
Special population-based interventions								
Glowa-Kollisch et al. (49)	8 (0.89%)	26–55	Identify and support people at risk Improve access and delivery of suicide care Teach coping and problem-solving skills	Plan for safety and follow-up after an attempt Create safer suicide care through systems change Support social–emotional learning programs	Includes Latinx	898	Quantitative; cohort-based study	Y
Hanes (50)	1 (25%)	21–24	Identify and Support People at Risk	Provide therapeutic approaches	Includes Latinx	4	Qualitative; case study	Y
Smigelsky et al. (52)	Unspecified	18+	Identify and support people at risk Teach coping and problem-solving skills Promote healthy connections Lesson harms and prevent future risks	Provide therapeutic approaches Support social–emotional learning programs Promote healthy peer norms Report and message about suicide safely	Includes Latinx	15	Quantitative; pre/post outcomes evaluation	Y
Tessier et al. (53)	12 (16%)	18–70	Identify and support people at risk Teach coping and problem-solving skills Promote healthy connections	Provide therapeutic approaches Support social–emotional learning programs Promote healthy peer norms	Includes Latinx	74	Quantitative; feasibility & acceptability study	Y
Zuromski et al. (54)	356 (9.7%)	18–55	Identify and support people at risk	Provide therapeutic approaches	Includes Latinx	3,649	Quantitative; observational proof-of-concept study design	Y

### Clinicians

We start this review with a summary of the suicide prevention approaches, interventions, and cultural modifications created for clinicians to address the needs of diverse patients, including Latinx persons. Clinicians include allied health professionals (e.g., community health workers, medical interpreters, medical assistants), counselors, physicians, nurses, and licensed clinical social workers. We include clinicians because it has been established that many people (30–38%) who die by suicide meet a clinician within a week before their suicide attempt (12, 13). Although suicide prevention requires structural- and community-level responses to prevent suicide, clinicians are influential in improving health behaviors and connecting patients to resources (14). We will commence with approaches, followed by interventions for clinicians, and we end with interventions that assessed both the clinician outcomes and Latinx persons’ suicidal behavior outcomes.

Almeida and colleagues (15) examined if education prepared future social work students’ knowledge and preparedness to identify suicide risk in their patients. Although this intervention was not geared toward Latinx-only clinicians, two clinicians (9%) self-identified as Hispanic/Latinx. The intervention was a pre- and post-survey design. The course included topics most important to social work clinicians such as screening, assessment, and management of suicidal patients. This study only assessed knowledge and preparedness in social work students through a pre- and post-survey and did not examine these outcomes in the students’ patients. The study did not explicitly mention a comparison group but did report statistically significant increases in knowledge (*t*[df = 21] = 4.79; *p* < 0.001), confidence (*t*[df = 17] = 8.55; *p* < 0.001), and preparedness (*t*[df = 20] = 7.28; *p* < 0.001) after the educational intervention.

Mattheiu et al. (16) provided a framework for outreach to public and private service providers who have access to the U.S. veteran population and who could serve as a gateway to needed Veterans Affairs and community-based suicide prevention services. Using a snowball sampling method, they recruited *n* = 70 Veteran’s Affairs and community-based providers. Participants then completed a survey focusing on five sections. The five sections were (1) organizational assessment, (2) provider demographics, (3) individual level factors, (4) exposure to suicide, and (5) awareness of suicide prevention resources. There was a 99% (*n* = 69) response rate. Ten percent of the participants identified as Hispanic/Latinx. The study did not have a comparison group. In conclusion, Mattheiu et al. report that veterans’ use of mental healthcare services was notable but there was an emphasis placed on using alternatives forms of help like education services and employment services. Although both Almeida and colleagues (15) and Mattheiu and colleagues’ (16) approaches included persons who self-identified as Hispanic/Latinx, none of these approaches reported the suicidal outcomes for the Latinx persons in among the clinicians’ patients after implementing the approach.

Reyes-Portillo and colleagues’ (17) study examined the effectiveness of integrating clinical decision support tools, like electronic health record (EHR) alerts, into the clinical care of youth at-risk for suicide. They tracked whether clinicians created a safety plan for youth flagged in the EHR for being at risk for suicide. There were 69 at-risk patients between the ages of 13–21 in the intervention period (*M* = 15.71; SD = 1.86; 66.7% female) and 64 (*M* = 15.38; SD = 1.93; 68.6% female) in the control period. Over 69% of the patients self-identified as Hispanic/Latinx. Most clinicians (82.5%) self-identified as non-Hispanic White persons. Logistic regression analyses indicated that patients in the intervention period were significantly more likely than patients in the control period to receive a safety plan (*B* = 3.64, CI: 1.63, 8.14, *p* < 0.01). Forty clinicians also completed a questionnaire assessing their satisfaction with the EHR alert, indicating moderate satisfaction (*M* = 3.01; SD = 0.63; range = 1.11–4.11). EHR alerts are associated with changes in clinicians’ behavior and improved compliance with best clinical practices for at-risk youth.

Unlike the Almeida and colleagues’ intervention (15), Waitzkin et al. (18) did assess patients’ outcomes in their clinician-based intervention with *promotoras* (bilingual, bicultural community health workers), who are known to improve health behaviors, outcomes, and quality of care. In a mixed-methods evaluation, Waitzkin and colleagues employed a survey and ethnography to assess the effectiveness of integrating *promotoras* in identifying depression care and addressing the structural sources of depression and suicide (e.g., underemployment, inadequate housing, food insecurity, and violence), among patients in New Mexico (NM) community health centers. Their intervention was motivated by the fact NM had the fifth highest suicide rates in the U.S. at the time of the intervention. They were one of the few interventions in this entire review that applied a conceptual framework to inform their intervention. One hundred twenty patients with depression (85% Hispanic/Latinx) were randomized to enhanced care plus the *promotoras* contextual intervention, or to enhanced care only. At baseline, 6 and 12 months of the intervention, there were no statistically significant differences between depression outcomes in patients in the contextual intervention versus those in enhanced care group (data not shown in article). Ethnographic results indicated that patients perceived *promotoras* as better listeners than primary care providers and found that they connected patients to bilingual and culturally appropriate services. Waitzkin and colleagues conclude that *promotoras* should be included in primary behavioral healthcare, especially to address the structural sources of mental illness in a primarily Hispanic/Latinx patient population.

Most clinician-based SP approaches, strategies and interventions that were created for clinicians serving Latinx patients focused on the CDC Suicide Prevention Strategy to *Identify and Support People at Risk*. In addition, Matthieu and colleagues (16) and Waitzkin and colleagues’ (18) interventions focused on the CDC SP Strategy to *Improve Access and Delivery of Suicide Care.*

### Late adolescents and young adults

Although the focus of this systematic review is to identify and describe suicide prevention approaches, interventions and cultural modifications for the U.S. Latinx adult population, we reviewed the following eight articles because they included non-minor (18+ years of age) late adolescents and young adults in their sample. Moreover, some of these measures and interventions are considered evidence-based suicide prevention strategies (e.g., Signs of Suicide) (10). We organized this section by first summarizing suicide prevention cultural modifications, then measures, and end with interventions.

Ford-Paz and colleagues (19) present a culturally tailored intervention for depression and suicide prevention for Latinx participants with an age range of 14–18 years for youth and 27–61 years for adults. The authors emphasized the need for a culturally tailored approach, which meant integrating strategies that resonate with the Latinx community’s cultural values, experiences, and social contexts. The intervention involves training community leaders in depression literacy and outreach, engaging in activities to increase social connections, and incorporating cultural enrichment. Participants were selected through purposive sampling among Latinx adult and adolescents in Chicago. Adult participants included youth program organizers, pediatricians, clergy, school personnel, and community health workers. Youth council members, health leaders, police volunteers, members of school health clubs, peer educators, tutors, and community activists were involved in the creation of the intervention. The authors also recommended ongoing, multimodal interventions that address stigmatized beliefs and attitudes about mental health help-seeking within both youth and their broader social networks. The comparison group was not explicitly described as a traditional experimental control group, and it was formative rather than comparative.

Ford-Paz et al. employed a community-based participatory research (CBPR) design, and the outcome of interest centered on combating mental health stigma, increasing knowledge about mental illness, and encouraging early mental health service utilization in ways that are meaningful within a Latinx cultural framework. For example, they emphasized educational campaigns and interventions that should be delivered by individuals whom the Latinx youth can identify with, such as those sharing similar ethnic and generational backgrounds, and incorporating testimonials to personalize and destigmatize mental health information, recognizing the importance of family within the community, and utilizing technology and social media to reach the target audience are also highlighted as key components. To achieve sustainable impact and reduce mental health disparities, the authors recommend adapting existing mental health programs and creating new initiatives with cultural relevance.

Silva and colleagues (20) report the multistage development and validation of the Spanish translation of the Interpersonal Needs Questionnaire (INQ-S-9). The primary outcome of this cross-sectional study design was the validation of the INQ-S-9, assessing its reliability, factor structure, and measurement invariance to evaluate its ability to measure the constructs of *thwarted belongingness* and *perceived burdensomeness* as proximal suicide risk factors across diverse Spanish-speaking populations, in both community and clinical settings. It is one of the few measures in this review that is informed by a theoretical or conceptual framework. Silva et al.’s sample consisted of college students from Spain (*n* = 1,016), Mexico (*n* = 239), and the United States (*n* = 337), Mexican psychiatric inpatients (*n* = 181), and U.S. Spanish-speaking adults (*n* = 104). The study explicitly compared the Spanish translation of the INQ across diverse Spanish-speaking populations. Between samples, there was good fit in the 9-item Spanish version of the INQ (INQ-S-9), as well as measurement invariance. The INQ-S-9 was consistent across nationalities and clinical severity levels. The INQ-S-9 can be a useful instrument for use in suicide risk assessment because it can supplement assessments that ask specifically and exclusively about desire and plans/intent for suicide through the assessment of proximal causes for suicidal desire.

Aseltine and DeMartino (21) examined the efficacy of the Signs of Suicide (SOS) program, a school-based suicide prevention intervention to deter suicidal behavior in teenagers. A brief screening for depression and other suicide risk factors is combined with suicide awareness education in the school-based SOS program. This quasi-experimental study involved over 2,000 students in high schools across Columbus, Ohio (OH) and Hartford, Connecticut (CT). The Hartford, CT sample included 53% females, with more 9th and 10th graders (35% and 30%), and 59% Hispanic/Latinx persons. The Columbus, OH sample had 48% females, with only 9th graders in the sample, and only 9% self-identified as Hispanic. This is one of few interventions in this review that uses a quasi-experimental design and randomizes participants into intervention and control groups between five schools in two cities. The intervention group received the Signs of Suicide (SOS) program, while the control group did not participate in the program until after the study evaluation. The exposure, or participation in the Signs of Suicide (SOS) program, included educational materials, a video, discussion sessions, and self-administered depression screening using the Columbia Depression Scale. The intervention’s primary outcome demonstrated success by significantly improving students’ knowledge and attitudes toward depression and suicide, accompanied by a marked reduction in self-reported suicide attempts, decreasing from 5.4 to 3.6% (−0.467, *p* < 0.05). However, for the Hispanic students, there were no significant decreases in suicide attempts (−0.193) or suicidal ideation (−0.245). Nonetheless, there was a statistically significant decrease in knowledge about depression and suicide after participating in the SOS Program among Hispanic/Latinx youth compared to the overall sample (overall: 0.689, *p* < 0.05; Hispanic/Latinx participants: −0.626, *p* < 0.05).

Under the CDC strategies of *Teach Coping and Problem-Solving Skills* and *Identify and Support People at Risk,* this study highlights the SOS program’s effectiveness in reducing actual suicide attempts and enhancing student awareness and attitudes toward mental health issues. There were fewer promising results among the Hispanic/Latinx students in the sample. The SOS suicide prevention program appears to provide short-term changes in high school students’ attitudes and behaviors about suicide. However, its emphasis on action over thought is credited for its success, although in this initial evaluation, SOS did not significantly alter suicidal ideation or help-seeking behavior in the youth. Limitations of this study include its short-term scope and the need for replication in diverse locations and in young adult-serving organizations, community colleges and vocational schools that have SOS licenses.

One of the few experimental designs in our sample of articles conducted a randomized controlled trial (RCT) to assess whether Dialectical Behavior Therapy (DBT), a structured approach that includes family involvement and teaching youth coping skills, was more effective than Individual and Group Supportive Therapy (IGST) in reducing suicide attempts, non-suicidal self-injury (NSSI) and self-harm (22). The intervention group received Dialectical Behavior Therapy (DBT), while the comparison group received Individual and Group Supportive Therapy (IGST). The exposure group was receiving DBT, with assessments conducted at baseline and at three, six, nine, and 12 months in a multisite randomized clinical trial study design. The primary outcomes measured were the frequency of suicide attempts, NSSI, and total self-harm episodes, assessed using the Suicide Attempt Self-Injury Interview, Schedule for Affective Disorders and Schizophrenia, and the Structured Clinical Interview for DSM-IV Axis II to assess personality disorders. There were 173 adolescent participants, across four academic medical center sites included 48 (27.5%) who self-identified as Hispanic/Latinx. Most DBT participants (97.7%) completed at least one post-baseline assessment and 91.9% of the IGST group completed one. There was greater participation in individual and group sessions among the DBT group compared to the IGST group, with 45.4% of DBT participants attending 24 or more sessions compared to only 16.1% for IGST. Of note is the fact that the DBT participants completed more treatment sessions, with an average of 19.97 individual sessions for DBT participants compared to 15.29 for IGST participants. The authors conclude that DBT could be more acceptable suicide prevention care; however, this was a predominantly female sample (94.8%). The study results may not be applicable to routine practice environments as this study was conducted under highly controlled conditions and by rigorously trained therapists.

Humensky and colleagues (23) created the Life is Precious (LIP) program, which aims to prevent suicide and address mental health issues in high-risk Latinx adolescent girls. The sample consisted of 107 Latina/x adolescents aged 11–18 years in New York City because 13% of Latina/x girls in grades 9–12 reported a suicide attempt in the past year (24). Among LIP’s main objectives, one was to provide a safe environment where adolescents can discuss key risk factors such as family conflict and acculturation, improve peer relationships, and enhance their academic performance. Among participants entering LIP, 18 individuals (17%) attempted suicide in their lifetimes. The study design was a multi-method study with community-based uncontrolled trial using retrospective data collection. The participants were exposed to the intervention, which included components such as family relationship support, academic assistance, creative expression activities, and wellness education, designed to address their mental health needs and reduce suicidal ideation.

The primary outcomes of the study showed that after completing six assessments during the 24-month program, no participant attempted or died by suicide, and the suicidal ideation reduced significantly with an average decrease of 2.3 points on their Suicidal Ideation Questionnaire (a valid and reliable measure for Spanish-speaking Latinx youth) (25) scores across 2 years. Participants who had a history of sexual abuse, tobacco use, or alcohol consumption showed greater improvements than those without such histories. Due to the program, depressive symptoms, anger, and post-traumatic stress were also greatly reduced, although family adaptability improved a bit, but not significantly. This could be because the program focused on acculturation, which led the participants to question how their families viewed gender roles and expectations.

Sekhar and colleagues (26) conducted a randomized clinical trial comparing the effectiveness of universal screening versus targeted screening in identifying and initiating treatment for adolescents at risk for suicide. Utilizing technology, the study enabled real-time identification of at-risk students through immediate electronic notifications to research staff, enhancing timely intervention. The researchers used data from the Screening in High Schools to Identify, Evaluate, and Lower Depression (SHEILD) trial, which compared universal screening using the Patient Health Questionnaire-9 (PHQ-9) to usual school practices of targeted referral. The population of focus in this study consisted of 12,909 adolescents aged 13–21 years from 14 Pennsylvania schools, including 20.8% who self-identified as Latinx, who were screened for suicide risk and randomly assigned to either universal or targeted screening. It is unclear what proportion of the sample was 18+ years of age.

As an outcome of the universal screening, there was a significant increase in the odds of identifying adolescents at risk for suicide, with adolescents being 7.1 times more likely to be identified (odds ratio (OR): 7.1, 95% CI: 5.7–8.8). Additionally, the likelihood of follow-up for those identified was enhanced, showing 7.8 times greater odds of requiring follow-up (OR: 7.8, 95% Cl: 4.6–13.1). Finally, the initiation of mental health treatment among identified individuals was also notably increased, with a fourfold increase in the odds of starting treatment (OR: 4.0, 95% Cl: 2.0–7.9). As a response to the US Preventive Services Task Force’s request for targeted versus general screening, SHIELD supports the effectiveness of suicide risk screening by increasing treatment initiation among identified adolescents. Additionally, the study stressed how technology can be used to identify at-risk students in real-time in primary care.

One intervention studied by Etter et al. (27), involved the implementation of a computer decision support system (CDSS) that integrated electronic medical records to screen adolescents for suicidality as part of their primary care visits. The screening was conducted using a pre-visit screener form administered on an electronic tablet, which included questions on suicidality, depression, and substance use. Clinicians were provided with a provider worksheet that offered specific follow-up recommendations to act upon. The sample consisted of 2,134 adolescent patients aged 12–20 years from a primary care clinic of which 305 (14.3%) self-identified as Latinx. Researchers found that just over 6% of participants reported suicidality in Indianapolis, where the intervention took place, with depressive symptoms emerging as a strong predictor of suicidality (OR: 16.66, *p* < 0.01), alongside female gender as a significant predictor (OR: 3.17, *p* < 0.01). A significant majority of the participants who were identified as “at risk for suicidality” received follow up (83%).

Primary care providers can effectively integrate suicide screening and follow-up guidance into existing CDSS. There are several limitations to the study, including relying on a single-item suicidality measure and being unable to measure the impact of screening and follow-up on patient outcomes. There is no explicit comparison group in this prospective cohort study design; instead, it assesses how the screening tool identifies suicidality and how effectively providers document and act on follow-up recommendations, without comparing these results to a control group or an alternative intervention. The primary exposure of using electronic tools in primary care was shown to be efficient at detecting and managing adolescent mental health issues, underscoring the potential to improve healthcare outcomes through electronic tools.

Sise and colleagues (28) administered a cross-sectional survey to evaluate whether a student-designed informational magnet with suicide warning signs and phone numbers for university counseling services and a national 24-h crisis line would increase students’ willingness to display the magnet prominently and their likelihood of using the listed services for themselves or others experiencing depression or suicidal thoughts. The magnet was distributed during the orientation period for first-year students and data were collected using a brief self-report survey. The magnet was intended to be kept in a visible location (e.g., on a refrigerator) as a constant reminder of the signs of suicide and available resources. Seven hundred and eight (*n* = 708) first-year, public university students (4.4% were 18+ years of age) were asked about their experiences with suicidal thoughts and behaviors, how they perceive stigma associated with seeking help, and how they feel about promoting and using an informational magnet. Overall, the primary outcome of this study was to evaluate the utility and acceptability of the informational magnet by raising awareness of suicide warning signs and promoting the use of available counseling and crisis resources. They found that 22% of students reported considering suicide due to recent thoughts, highlighting the urgent need for effective interventions targeting young adults. Over half of the students (59%) displayed the informational magnet in a visible location, and 63% contacted a service listed on the magnet if they knew someone in need. While gender did not affect students’ response, those who self-identify as Latinx were more likely to respond favorably than non-Hispanic White persons (74% vs. 57%; *p* < 0.01).

Frick, Butler, and deBoer (29) discussed a universal suicide screening and care program implemented at Loyola University Chicago’s student primary care setting to increase student and staff awareness, support, and education about suicide prevention. What makes this intervention unique is its multifaceted approach by providing an early detection screener through the Annual Suicide Behaviors Questionnaire but also provides a systematic support structure to students. This includes an EMR (Electronic Medical Record) template that streamlines documentation and monitoring, safety alerts for immediate response to identified risks, referral tracking to ensure continuous care, and simulated training sessions to enhance staff preparedness and responsiveness. The Universal Suicide Screening Program is a three-month pilot study employing a pre/post outcomes evaluation design rather than a traditional control group to demonstrate the feasibility and effectiveness of the program in a university primary care setting by identifying at-risk students across diverse demographics and integrating diverse clinical staff. The sample consisted of 1,607 college students seeking care at the university health services during spring 2018, between 18 and 22 years old. Chi-square tests assessed documentation, safety alerts and mental health referral changes. The primary outcomes included the percentage of students screening positive for suicide risk using the Suicide Behaviors Questionnaire-Revised (SBQ-R), improvement in documentation consistency, increased use of EMR safety alerts, and the number of mental health referrals and appointments resulting from the screening program. Paired *t*-tests evaluated staff learning outcomes. Male suicidality rates were slightly lower than females, with males at 11.58% and females at 12.69%. There was a consistent trend in suicidality across academic levels but differences in self-identified race/ethnicity, with American Indian students having the highest rate. Latinx students reported a positivity rate of 14.22%, which was comparable to the other racial/ethnic groups.

Rivero and colleagues (30) discuss the development, implementation and evaluation of the CARE Net program, an early intervention suicide prevention program designed to respond to residential college students manifesting risk for suicide, such as connecting the student to vital services and support networks. This was a case study design evaluating the implementation and outcomes of the CARE Net program for referred students. This study does not include a comparison group. The sample consisted of 108 undergraduate residential students at risk for suicide and were connected to essential mental health services through this program. These programs provided a multidimensional assessment and referral system post-crisis, as opposed to involuntary medical withdrawal strategies that may not provide the same level of support or opportunity for recovery and reintegration into the university. University staff and mental health professionals evaluated students’ suicide risk, developed intervention plans, and involved students’ support networks to suicidality. The program facilitated access to support services and created a personalized prevention and intervention plan for each student. The CARE Net appeared to successfully support students at risk of suicide by improving retention rates and recovering their GPA following the intervention (*t*(43) = 2.13, *p* = 0.04). The Universal Screening at Loyola and CARE Net’s findings are limited because their implementation and evaluation are limited to one university and they lack long-term follow-up with students, making it difficult to determine the most effective components of the intervention, as well as when to intervene and establish causality.

De Luca and colleagues’ (31) study explores the patterns of help-seeking behavior among college students, particularly focusing on those with recent suicidal ideation. The sample comprised of 26,292 college students from 73 four-year institutions across the United States during the 2010–2011 academic year. The study utilized a cross-sectional survey design that compared students with recent suicidal ideation (in the last 12 months) to those without recent suicidal ideation. It assesses the effectiveness of online and traditional help-seeking methods, revealing that while both methods are utilized, with females and younger students being more likely to engage in help-seeking behaviors. However, those with recent suicidal ideation were less likely to seek any help. The study suggests that online resources could potentially bridge the gap for those reluctant to seek traditional help but emphasizes that traditional resources remain more effective for the sample studied. The research highlights the importance of online help-seeking as a complementary approach to traditional methods in suicide prevention strategies.

Chesin and colleagues (32) conducted an observational study that examined the association between viewing the Netflix series “13 Reasons Why,” a popular psychological thriller about a young woman, Hannah, who left information about her 13 reasons why she died by suicide. The authors employed a cross-sectional survey design, comparing students who watched “13 Reasons Why” (63.8%) to those who did not. The authors asked college students the severity of their suicidal ideation, their behavior risk, their stigma, and their knowledge about suicide while this show was at its height. The sample consisted of 818 college students, ages 18–25, with 64% of participants having watched the Netflix series. Chesin et al. found that there was no significant association with suicidal ideation severity or behavior risk, suggesting that the statistical power for detection was limited. There was a significant association between viewing and knowing about suicide risk factors, particularly among those who did not have personal experience with suicide, and it also resulted in a reduction in the stigma, ideation, and behavior related to suicide, which also aligns with the CDC’s *Lessen Harms and Prevent Future Risk* strategy. The authors suggest that the series could be considered a psychoeducational tool for suicide, it is important to explore its impact on suicide behavior risk in more detail.

Similarly, Vargas and colleges (33) employed Critical Consciousness Theory to co-create telenovela films with Latinx LGBTQ+ youth, depicting their experiences of social isolation. They employed a community-engaged qualitative research design, which did not include a comparison group. While the study did not directly report the intervention’s impact on suicide, it identified factors contributing to and protecting against isolation. The study underscores the importance of core values and intersectional identities in fostering solidarity and challenging oppressive systems and provides insight to improve environments for Latinx LGBTQ+ individuals to reduce suicide risk.

In summary, most of the SP approaches, strategies, and interventions for late adolescents and young adults do not exclusively focus on Hispanic/Latinx persons, except for Ford-Paz and colleagues’ culturally tailored mental health intervention (19), Silva and colleagues’ translation of the INQ (20), Humensky and colleagues’ *Life is Precious* intervention (23, 24), and Vargas and colleague’s telenovela creations (33). Under the CDC Suicide Prevention Strategies, the SP interventions for late adolescents (*n* = 8) primarily sought to reduce suicidal behavior by *Identifying and Supporting People at Risk* (*n* = 6) (19, 21–23, 26, 27) *Promoting Healthy Connections* (*n* = 3) (19, 24, 28), and *Teaching Coping and Problem-Solving Skills* (*n* = 3) (21, 23, 24). The SP interventions for young adults (*n* = 6) were more multifaceted because they often sought to reduce suicide behavior by *Identifying and Supporting People at Risk* (*n* = 3) (20, 29, 30), *Improving Access and Delivery of Suicide Care* (*n* = 3) (29, 31, 33), *Creating Protective Environments* (*n* = 2) (29, 30), and *Promoting Healthy Connections* (*n* = 2) (29, 30). Many of the late adolescent SP interventions discussed here were implemented in secondary schools and community-based settings, while those for young adults were implemented in university settings like on-campus healthcare facilities and college dormitories. The culturally tailored interventions were based on convenience samples, and it is difficult to know from the way the samples were described if the participants represented diverse Hispanic/Latinx sub-groups. Most of these interventions were evaluated comparing pre- and post-intervention scores on self-reported psychometrics, in some cases between a control and intervention group. None of the analyses included effect modifications of demographic characteristics or clinical conditions between the intervention and suicidal ideation, attempts and deaths.

### Adults

Of the 34 articles in our final analysis, eight focus on measures and interventions to prevent suicide among adults outside university settings and later in life. Three studies specifically target Latinx adults, while the remaining five include some percentage of Latinx individuals. This section first describes measures to assess suicide risk (11, 34, 35), followed by suicide prevention interventions (36–40).

Kimbrel and colleagues (11) developed and validated the Durham Risk Score (DRS), a suicide risk checklist/calculator aimed at identifying individuals at risk for suicide attempts within 1–3 years. The sample was drawn from three cohorts: the National Epidemiologic Survey on Alcohol and Related Conditions, the Assessing and Reducing Post-Deployment Violence Risk Study, and the Veterans After-Discharge Longitudinal Registry. The quantitative study included a total of 35,654 participants, with 17,630 used to develop the DRS and 18,024 involved in its validation. Latinx individuals comprised approximately 18% of participants in both phases. The outcome of interest was the prediction of suicide attempts, measured through area under the curve (AUC) metrics. The study utilized a prospective cohort design to ensure the robustness of findings. The DRS demonstrated strong predictive validity in both the development cohort (area under the curve [AUC] = 0.91) and the validation cohort (AUC = 0.92). Notably, 82% of future suicide attempts occurred among those with DRS scores in the top 15%. While no formal comparison group was included, the DRS’s performance was evaluated across diverse demographic subgroups. The tool also performed well across various demographic subgroups, including women (AUC = 0.91), men (AUC = 0.93), Black persons (AUC = 0.92), White persons (AUC = 0.93), Hispanic/Latinx persons (AUC = 0.89), veterans (AUC = 0.91), lower-income individuals (AUC = 0.90), younger adults (AUC = 0.88), and LGBTQ individuals (AUC = 0.88). Kimbrel and colleagues concluded that the DRS shows promise in enhancing clinicians’ ability to identify individuals at risk of suicide attempts.

Similar to the DRS, this next study’s instrument, the eC-SSRS, did not involve treatment, but another tool to assess the risk of suicide. Gwaltney and colleagues (34) validated a tablet-based version of the electronic Columbia-Suicide Severity Rating Scale (eC-SSRS) against an interactive voice response (IVR) version. The quantitative study used a randomized crossover-equivalence design, and the primary outcome was agreement between the tablet and IVR versions. The eC-SSRS was designed to assess severe lifetime and recent ideation, suicide attempts, interrupted and aborted attempts, preparatory behaviors, and non-suicidal self-injurious behavior. Gwaltney et al. sought to facilitate the prospective monitoring of suicidal ideation and behavior in clinical settings. The study sample consisted of 86 participants, including 58 hospital patients and 28 hospital employees, with Latinx individuals comprising approximately 5% of the sample. Racial distribution was consistent between case (eC-SSRS) and control (IVR) groups. The findings demonstrated high validity, reliability and high agreement between the tablet and IVR versions across measures related to suicidal ideation and behavior. The authors concluded that the tablet-based eC-SSRS is both valid and reliable for assessing suicidal ideation and behavior, though the study did not differentiate usage patterns by race/ethnicity.

Silva and colleagues (35) used a prospective observational study examining feasibility, adherence, and clinical insights gained from using smartphones for remote Ecological Momentary Assessment (EMA) of suicidal ideation and risk factors among Latinx adults who primarily speak Spanish, compared to standard paper-pencil measures to evaluate effectiveness in capturing variability in suicide risk. The sample included 16 adult psychiatric outpatients, recruited from a Spanish-language outpatient mental health clinic serving a predominantly low-income Puerto Rican community (76%). Most participants (81.3%) were female, with an average age of 43.75 years. Participants completed EMA smartphone reports, including assessments of suicidal ideation, four times daily over 14 days. EMA data indicated significant variability in suicidal ideation and risk factors throughout the day, with many participants experiencing notable changes within short periods. The outcome demonstrated strong adherence to EMA, with participants completing an average of 74.05% of the EMA survey instances. Follow-up qualitative interviews assessed the acceptability of study procedures. The findings suggest that observational EMA via smartphones is feasible and capable of providing valuable clinical insights in an understudied and linguistically isolated population, specifically among primary Spanish-speaking outpatient psychiatric patients prone to suicide.

Constantino and colleagues (36) assessed the impact of cultural congruence (CC) between mental health providers and their clients on mental health treatment outcomes, including suicidal behavior, among older Latinx adults. The study design was a subset analysis within a larger multisite observational research study, focusing on treatment outcomes across different service delivery models. The sample consisted of 272 older Latinx adults (ages 65–94) who were part of the Primary Care Research in Substance Abuse and Mental Health for the Elderly (PRISM-E) study, a multisite national research initiative. Participants were drawn from 3 of the 10 PRISM-E study sites. The exposure was cultural congruence, measured through a CC index that assessed the alignment between clinic cultural competence and the importance of culture to clients. Cultural congruence was associated with reduced symptoms of depression and suicidality, as well as improved physical functioning. The comparison involved variations in CC levels across clinics and treatment modalities, particularly integrated primary care versus referral-enhanced specialized mental health services. The outcome demonstrated that higher cultural congruence predicted improved mental health treatment outcomes, including reductions in depressive symptoms, suicidality, and anxiety, alongside better physical functioning. Constantino et al.’s evaluation suggests that a referral-enhanced specialized model of mental health services may be more effective for older Latinx adults experiencing suicidality, highlighting the role of cultural congruence in fostering positive treatment outcomes.

Goodsmith and colleagues (37) described the development and implementation of a Suicide-Risk Management Protocol (SRMP) in two under-resourced, predominantly Black and Latinx communities in Los Angeles. The study design was a longitudinal observational study to evaluate the feasibility and impact of the SRMP. The sample included 1,018 adults exhibiting moderate to severe depressive symptoms, with 48% identifying as Black and 40% as Latinx. The intervention was the SRMP, developed using a community-partnered participatory research framework. The protocol included rapid, high-quality outreach, assessment, and brief counseling by a licensed clinician via phone, along with arrangements for expedited mental health intake through local outpatient clinics for individuals not already in treatment. The comparison was between community-partnered SRMP services and the absence of such structured suicide risk management approaches in under-resourced settings. The study followed participants over 4 years, during which no known suicides occurred among the sample. The outcome of interest was participant safety, measured by the absence of suicide deaths and the effective referral to and utilization of mental health services. The workgroup responsible for developing the SRMP included diverse community stakeholders, but the inclusion of Latinx representatives in SRMP’s development was not indicated. The authors concluded that SRMP can be effectively implemented in racially marginalized communities, highlighting their potential for broader application in clinical settings. This is the first study to address the benefits of SRMPs in racially marginalized communities and indicates the future potential of their implementation in clinical services.

Arias and colleagues (38) explored the implementation of crisis hotlines as a safety protocol within the Emergency Department Safety Assessment and Follow-up Evaluation (ED-SAFE) study. This was a quasi-experimental clinical trial conducted across eight emergency departments in the United States. The sample included 874 adults who had contemplated suicide in the past week or experienced recent suicide attempts between 2009 and 2013. Of these, 135 participants (16%) were transferred to a crisis hotline, and 131 (97%) completed a crisis counseling call. The sample was predominantly Non-Hispanic White (80%), with 11% identifying as Latinx. The intervention examined was the Boys Town National Hotline, which provided 24/7 access to trained counselors for emergency response during follow-up assessments when on-site mental health clinicians were unavailable. The comparison involved variations in use of the hotline versus non-transfer to assess differences in safety outcomes. The hotline used a standardized evaluation process that included problem identification, exploring options, and creating a plan to encourage follow-through and referrals. Demographic analysis revealed that sex (*p* = 0.09) showed a near-significant difference, with more females receiving crisis counseling (67%) compared to males. Age, race, and ethnicity did not show significant differences between those who received and did not receive counseling. The outcome demonstrated the effectiveness of the hotline, evidenced by a high call completion rate (97%) and the absence of any immediate adverse events reported during crisis interventions. The authors concluded that crisis hotline interventions hold potential as a safety mechanism for high-risk populations in diverse healthcare settings.

Dueweke and Bridges (39) conducted a randomized controlled trial during community events to investigate the impact of brief, passive psychoeducation on suicide literacy among first-generation Latinx immigrants. Seventy-eight (*n* = 78) first-generation Latinx immigrants aged 21–70 years were recruited from community events in a mid-southern U.S. region. Participants were randomly assigned to the control or experimental condition using odd-numbered (control group) or even-numbered (experimental group) questionnaire packets from a shuffled stack. Before the intervention, all participants provided demographic information and answered questions about suicide literacy, with no initial differences between the groups. The intervention involved brief, passive psychoeducation through a brochure developed by the National Institute of Mental Health (NIMH), provided in English and Spanish. Researchers compared literacy, stigma, and help-seeking attitudes between two groups: one receiving suicide psychoeducation from the NIMH brochure, and the other receiving psychoeducation on walking. The suicide psychoeducation group scored higher on a literacy quiz (*M* = 82% vs. *M* = 76%, *t*(76) = 2.16, *p* = 0.03). However, there were no significant differences in non-stigmatizing attitudes (*M* = 11.00 vs. *M* = 11.76, *t*(74) = −1.07, *p* = 0.29) or help-seeking attitudes (*M* = 12.33 vs. *M* = 11.56, *t*(76) = 1.40, *p* = 0.17). The authors did not assess whether passive psychoeducation reduced suicidal ideation, attempts, or deaths by suicide.

McCourt and colleagues (40) investigated the effects of state background check policies on firearm-related mortality in four U.S. states: Maryland, Pennsylvania, Connecticut, and Missouri. The study design was a quantitative observational study, employing synthetic control methods to analyze policy impacts over an extended timeframe. The study analyzed the impact of comprehensive background check (CBC) laws and handgun purchaser licensing laws on homicide and suicide rates, stratified by firearm involvement, using annual data from 1985 to 2017. The exposure included CBC laws that required background checks at the point of sale and handgun purchaser licensing laws, which required applicants to obtain a license through state or local law enforcement. The comparison assessed outcomes in states without these policies, using synthetic control methods to estimate the counterfactual (what outcomes would have been in the absence of the laws). The study found no consistent relationship between CBC laws and mortality rates in Maryland and Pennsylvania. However, Connecticut’s handgun purchaser licensing law was linked to a 32.8% decrease in firearm suicides, while Missouri’s repeal of handgun purchaser licensing laws was associated with a 23.5% increase in firearm suicides. The outcome demonstrated that purchaser licensing laws, when combined with CBC requirements, were consistently associated with lower firearm-related suicides and homicides. The authors emphasized the need for enhanced enforcement of CBC laws, promotion of compliance, and additional measures like purchaser licensing laws to achieve meaningful reductions in firearm-related mortality.

Our review of these eight suicide prevention research papers reveals a diverse array of approaches, assessments, and interventions, ranging from crisis hotlines to culturally congruent mental health treatments for adults. These studies highlight key CDC strategies for reducing suicide, particularly to *Improve Access and Delivery of Suicide Care* (*n* = 6) (11, 34–38) and *Identify and Support People at Risk* (*n* = 5) (11, 34, 35, 37, 38). Additionally, one paper includes strategies to *Promote Healthy Connections* (37) and another focuses on *Teaching Coping and Problem-Solving* (39). Among these interventions, only one addresses suicide prevention at the structural level by *Lessening Harms and Prevent Future Risk* (40). Notably, only three of the eight studies specifically target Latinx adults, including one aimed at first-generation immigrants and another at Spanish-speaking individuals.

### Families

Several studies have demonstrated that supportive familism enhances help-seeking behaviors and reduces internalizing symptoms among Latinx adolescents and young adults, including reducing the risk of suicidal ideation (41–43). This section details three from our final analysis, focusing on family-based interventions aimed at decreasing suicidality in Latinx youth.

Diamond and colleagues (41) reported on a randomized controlled trial (RCT) that compared Attachment-Based Family Therapy (ABFT) and Family-Enhanced Non-Directive Supportive Therapy (FE-NST) for suicidal adolescents aged 12–18 years. The population of focus was suicidal adolescents, of which the study included 129 recruited from emergency departments, inpatient psychiatric hospitals, mental health agencies, and schools. Participants were racially and ethnically diverse, with 15.5% identifying as Latinx. The interventions compared were ABFT, focusing on resolving attachment disruptions between adolescents and their parents, and FE-NST, which aimed to build a supportive relationship among the adolescent, family, and therapist. Participants were randomly assigned to ABFT (*n* = 66) or FE-NST (*n* = 63) for a 16-week treatment period. The outcomes included a reduction in suicidal ideation, with ABFT showing an estimated change of −5.40 (se = 0.50) and FE-NST −4.87 (se = 0.50) on the Beck Depression Inventory II scale. Both interventions led to significant reductions in suicidal ideation and depressive symptoms, with no significant differences between them (*F* (1,127) = 0.11, *p* = 0.74). However, the study’s hospital and university setting may limit generalizability, and some participants required extended or additional treatments. This RCT highlights the comparable effectiveness of both interventions in reducing suicidal ideation and depressive symptoms in a diverse adolescent population.

Project Wings Home Visits is a school-based health promotion intervention designed to overcome barriers faced by Latinx parents in supporting the mental health and academic success of their adolescent children (42). Created through a community-based participatory research (CBPR) approach, Project Wings utilized community health workers (CHWs) as the intervention to provide home-based outreach, education, and connections to resources such as counseling and social support. The initiative involved collaboration among Saint Paul Public Schools, the University of Minnesota School of Nursing, and West Side Community Health Services, focusing on improving mental health outcomes for Latinx adolescents and their families. This was a descriptive, concept paper providing details about the development and implementation of Project Wings. The sample consisted of Latinx parents and their adolescent children, particularly those served by Saint Paul Public Schools and West Side Community Health Services. The comparison was the absence of direct home-based CHW outreach, which traditionally limited Latinx parents’ engagement with mental health and academic resources. The outcome emphasized the feasibility and acceptability of CBPR-driven interventions for Latinx families, though specific mental health or academic success metrics were not reported. While the study highlighted the importance of partnerships in addressing complex mental health challenges, it did not evaluate the intervention’s effectiveness in reducing suicidal behaviors among parents and youth.

Like Project Wings, Vidot and colleagues (43) presented a culturally tailored family-based intervention, Familias Unidas, designed to prevent and reduce problem behaviors, substance use, and risky sexual behaviors in Latinx adolescents. This was a randomized controlled trial with follow-up assessments at baseline, six, 18 and 30 months, achieving retention rates above 87%. The sample consisted of 746 Hispanic eighth-grade adolescents and their primary caregivers, with 88% of parents born in Spanish-speaking Latin American countries and 55% of adolescents born in the United States. The intervention, Familias Unidas, emphasized teaching coping and problem-solving skills through family-centered, multiparent groups, private parent sessions, and family sessions focused on improving family functioning and communication. Participants were randomly assigned to either Familias Unidas (*n* = 376) or Prevention as Usual (*n* = 370), a standard school-based HIV risk reduction program delivered by teachers. Vidot et al. evaluated the effects of each program fon problem behaviors, including suicidal ideation and attempts. The study found no significant differences between conditions in suicidal ideation (*b* = −0.129, *p* = 0.130) or suicide attempts (*b* = −0.024, *p* = 0.744). However, a significant interaction effect was observed, where Familias Unidas reduced suicide attempts among adolescents with lower baseline parent–adolescent communication (interaction effect: *b* = −0.01, *p* = 0.01). The outcome highlighted the importance of parent–adolescent communication in moderating the effectiveness of the intervention on suicidal behaviors.

These three Latinx family-based interventions use distinct methodologies to evaluate their impacts on suicidal behaviors, emphasizing cultural sensitivity and community engagement. Aligned with CDC strategies for reducing suicide, these studies primarily focus on *Teaching Coping and Problem-Solving Skills* (*n* = 3) (41–43) and *Identifying and Supporting People at Risk* (*n* = 2) (41, 42). Additionally, Project Wings Home Visits focuses on *Improving Access and Delivery of Suicide Care* and *Promoting Healthy Connections* (42). These studies highlight the importance of family-centered interventions in mitigating suicide risk among Latinx youth. Familias Unidas underscores the pivotal role of family dynamics in fostering positive outcomes, showing greater impact on youth’s suicidal behavior when they start with less parent-youth communication. All studies emphasize the necessity for extended treatment durations, indicating that effective suicide prevention requires ongoing, long-term efforts.

### Special populations

We have a section on special populations to acknowledge the ways in which intersectionality (44) or multiply marginalized positions, can make segments of the Latinx population more vulnerable to suicidal behavior. This section will discuss suicide prevention (SP) approaches, strategies and interventions for Latinx adults who are or were incarcerated and veterans and active military. These two populations are especially vulnerable to suicidal behavior because their suicide rates are higher than the civilian population. According to 2014 National Violent Death Reporting System, the suicide rate for persons in jail or prisons ranged between 14 and 50 deaths per 100,000, which was higher than the U.S. general rate of 12.9 that same year. In addition, incarcerated persons often die by suicide within less than 1 week of incarceration (45). Similarly, veterans only comprise 8% of the U.S. population, but they account for ~13.8% of all suicide deaths among U.S. adults (46). In 2018, the Veteran suicide rate was 1.5 times that of non-Veteran adults even after adjusting for age and gender (47). Meanwhile, formerly incarcerated population (i.e., re-entry population) “are greater than seven times more likely to die by suicide than the general population (48).” Below we discuss the few SP strategies that included Latinx adults in their sample, as none were exclusively created to mitigate suicidal behavioral among re-entry or veteran and active military Latinx adults.

Glowa-Kollisch and colleagues (49) and Hanes (50) had two distinct SP interventions for incarcerated persons. Glowa-Kollisch et al. implemented a cohort-based study evaluating a unique mental health program, Beyond the Bridge (BTB), for incarcerated men (ages <=25 and > =56) in the New York City Jail System between 2010 and 2011. Thirty-three (8%) of the participants self-identified as Hispanic/Latinx inmates. BTB provides diverse modalities of cognitive behavioral therapy in jail mental observation units. The program included group therapy and individual encounters with social workers, psychologists, psychiatrists, and discharge planners. The rationale for this new approach was to use an inpatient psychiatric center treatment model (versus a medication regimen only, i.e., control group) to treat psychiatric conditions, while incentivizing inmates with commissary dollars or paid peer leadership roles. The outcomes they examined related to suicide were reduction in time spent on suicide watch and incidents of self-injurious behavior. To ensure that there were no significant clinical and demographic differences between treatment and control groups the authors used propensity score matching prior to the intervention and computed a Mantel–Haenszel rate ratio for each outcome to account for stratification resulting from propensity score matching. There were statistically significant smaller rate ratios for number of days on suicide watch for men in the treatment versus the control groups (rate ratio [RR] = 0.72; 95% [CI] = 0.59, 0.89) and self-injurious behaviors ([RR] = 0.87 95% [CI] 0.31, 2.46). Increased program participation was not associated with decreased risk of suicide watch days or self-injurious behaviors. Issues with the evaluation include that there was inconsistent correctional staff participation, the participants could not be randomized into the groups given the setting, and results were not reported by racial-ethnic groups.

Hanes (50) described an art therapy technique, road drawing, in a case study of four inmates on suicide watch. There was no comparison group. Road drawings were used during the clinical interview with an art therapist to evaluate an inmate’s risk for suicide in a Midwestern County jail. The participants were 21–24 years of age with one of the four participants identifying as a Latinx man. The author described how road drawings can be used to describe personal history, mental and emotional states, and behavioral patterns that may put a person at risk for suicide. Despite the small sample size, Hanes concluded that road drawing provides a quicker way for clinicians to evaluate suicidal ideation and threat of harm in the incarcerated population. There has been no evaluation of the road drawing art therapy technique at a larger scale at this or other correctional facilities, but it could be a technique integrated into other carceral clinical interventions. Given its small sample size, lack of comparison group and objective measures, it is difficult to discern the generalizability and efficacy of road drawing to reduce suicidal ideation, attempts and deaths among incarcerated inmates, much less Latinx incarcerated persons.

The last three interventions focused on veterans and active military service members but included Hispanic/Latinx participants in the study sample. Currently, 18.4% of active-duty members of the U.S. military self-identify as Hispanic/Latinx (51). Smigelsky and colleagues (52) described and evaluated the “Reclaiming Experiences and Loss” or REAL program, which is group therapy administered by a social worker and a chaplain from the Eastern Oklahoma Veterans Affairs (VA) Health Care System to mitigate the mental health effects of moral injury, including post-traumatic stress disorder (PTSD), depression, and suicidality. It consisted of group therapy over 12 weeks with the veterans completing individual worksheets before each session. Four cohorts of 19 participants (two to six participants in each participant) were recruited and participated between September 2017 and January 2019. The authors did not have a comparison group or collect much demographic information from the participants. However, we know from the case studies presented that at least one participant was a 54-year-old Latinx male.

The participants were given the PTSD checklist and the Patient Health Questionnaire-9 (PHQ-9) pre- and post-REAL to assess changes in PTSD and depression symptomatology. The PHQ-9 includes one item on suicidal ideation. Complete data are available from 15 of the 19 participants and reveal that the sample had a mean PTSD score of 60.0 (SD: 11.5) pre-group and 30.4 (SD: 13.6) post-group. The sample had a mean PHQ-9 score of 18.3 (SD: 6.1) pre-group and 5.5 (2.5) post-group. Ten veterans who endorsed suicidal ideation on the PHQ-9 item on their pre-group measure (i.e., *thoughts that they would be better off dead or of hurting themself*), none endorsed suicidal ideation in the previous 2 weeks when completing post-group survey. Although REAL appears to be a promising intervention to mitigate suicidal ideation in veterans suffering from moral injury, there may be selection bias issues and a small sample size to generate definitive conclusions about its effectiveness. Nonetheless, the REAL Program merits a larger clinical trial with a larger sample racial-ethnic and gender diverse veterans as well as veterans from distinct military branches and geographic locations.

Tessier and colleagues (53) describe a holistic and multimodal psychiatric intervention for veterans in the VA of Greater Los Angeles and Long Beach Health. The intervention was the Therapeutic Lifestyle Change (TLC) program designed for veterans with serious mental illness. It involved four biweekly classes focused on different aspects of lifestyle change (biological, psychological, social, and spiritual) and one-on-one coaching to help participants maintain these changes. The study was piloted first to assess acceptability and feasibility, and then a second time to assess efficacy of reducing psychiatric symptoms, weight and healthy lifestyles among veterans for 9 weeks. The sample consisted of 78.2% males and 21.8% females in Pilot Study 1 (*n* = 55), and 84.2% males and 15.8% females in Pilot Study 2 (*n* = 19). In Pilot Study 1, the sample was diverse with African Americans/Black persons (25.5%), Asian/Pacific Islanders, (1.8%), non-Hispanic White persons (58.2%), and Hispanic/Latinx persons (12.7%). In Pilot Study 2, the sample consisted of Asian/Pacific Islanders (5.3%), non-Hispanic White persons (63.2%), Hispanics/Latinx (26.3%), and 5.3% identifying as “Other” race/ethnicity. The average age was 57.4 years. Participants had various diagnoses including schizophrenia, schizoaffective disorder, bipolar disorder, PTSD, and other mental illnesses.

Researchers found that engagement in more TLC was associated with higher ratings of quality of life as measured by the Biopsychosocial Spiritual Wellness Self-Appraisal Scale (BPSS). For each additional TLC practiced, there was an average increase of 1.52 points on the BPSS scale. The authors listed as anticipated improvements lower suicide risk, however, they did not specifically report results on the improvement of psychiatric or suicidal risk factors or behaviors. The participants did experience improvements from weeks 1 to 9 in their physical health and significant increases in the WHOQOL-BREF psychological domain (17.2–18.9) (*F*_(1,19)_ = 4.4, *p* = 0.05).

Zuromski and colleagues’ study (54) was done in the context that the U.S. Department of Defense wants to require annual suicidal ideation (SI) screening among its active military. The authors report a multistep process to improve in-depth suicide risk assessments among active-duty U.S. Army soldiers. This was a prospective, observational proof-of-concept study design, that took place between 2010 and 2014, examining whether a brief structured questionnaire administered to US Army soldiers can help target in-depth suicide risk assessments by assessing soldiers with self-reported lifetime suicidal ideation (SI) and have an administratively recorded suicide attempt (SA). The authors utilized data from the Army Study to Assess Risk and Resilience in Servicemembers (Army STARRS). This was a probability sample of 17,462 active Regular Army, National Guard, and Army Reserve soldiers worldwide, except for those deployed or in basic training. The final sample (N = 3,946) consisted of mostly males (80.5%) and had a median age of 29 years (range, 18–55 years). Although most were non-Hispanic White persons (69.4%), 9.0% were Hispanic/Latinx persons. Sixty-five respondents had administratively recorded nonfatal SAs between survey response and December 2014.

The primary outcome was administratively recorded nonfatal SAs between survey response and December 2014. Significant risk factors were SI recency (odds ratio [OR], 7.2; 95% CI, 2.9–18.0) and persistence (OR, 2.6; 95% CI, 1.0–6.8), positive screens for mental disorders (OR, 26.2; 95% CI, 6.1–112.0), and Army career characteristics (OR for junior enlisted rank, 30.0; 95% CI, 3.3–272.5 and OR for senior enlisted rank, 6.7; 95% CI, 0.8–54.9). The respondents with highest estimated risk accounted for 39.2% of subsequent SAs. Zuromski and colleagues found that a composite risk index, developed from a relatively small number of self-report survey risk factors, could identify a small proportion of soldiers with elevated risk for subsequent suicide attempts. There is room for improving targeted in-depth suicide risk assessments for active Army servicemembers.

None of the approaches and interventions for incarcerated persons and veterans and/or active military were Latinx-specific, and primarily addressed the CDC SP Strategies *Identifying and Supporting People at Risk* (*n* = 4) (49, 50, 52, 54), *Promoting Healthy Connections* (*n* = 2) (52, 53), and *Teaching Coping and Problem-Solving Skills* (*n* = 2) (49, 53). The rate of suicide is higher post-carceral release and highest among women (46). So, there should be suicide prevention approaches and interventions that target these formerly incarcerated Latinx persons. What the re-entry population and the veteran population share is that they both emerge from a total institution, or “… a place of residence and work where a large number of like-situated individuals [are] cut off from the wider society for an appreciable period of time” (48). Although there were behavioral and community-based interventions for veterans (52, 53), there were none for active military. In this section, some interventions were rigorously evaluated, including one cohort-based study with a control group (49), but it was not a representative sample of incarcerated persons. None of these interventions addressed the multiple marginalized positions that Latinx adults face such as living as marginalized racial-ethnic groups, sexual minorities and those with lower income.

## Discussion

The goal of this scoping literature review was to synthesize suicide prevention (SP) approaches, measures, interventions and cultural modifications of existing SP interventions for the U.S. adult Latinx population between 2000 and 2024. Ten of the reviewed SP approaches, measures and interventions were created exclusively for the U.S. Latinx population, but only four were created specifically for Latinx adults outside of college settings. Here we will evaluate the theoretical and methodological rigor of these SP interventions created from rigorous research and their efficacy in reducing suicidal ideation, attempts, and deaths by suicide. In each section, we make recommendations addressing the gaps in the existing literature on suicide prevention approaches, culturally adapted strategies, and interventions, but also how they are can address diverse disciplinary perspectives and the multiple levels by which society, community and individuals can shape suicidal ideation.

Very few of the reviewed articles applied a conceptual or theoretical framework to inform their SP approach or intervention. Of the ones that did, Silva et al. (20) applied the interpersonal theory of suicide and Waitzkin and colleagues’ (18) *promotora* intervention was informed by the social contextual factors of the biopsychosocial model. However, the two predominant disciplinary approaches to suicide are psychology and sociology. Psychological approaches to suicide attribute suicide to individual, mental and emotional pathologies, such as untreated mental illness, substance abuse, and intense emotional suffering (6). Meanwhile, sociological explanations for suicide, often adapted from Emile Durkheim’s theory of suicide (55), are perceived as a social problem in which society has diverse and conflicting norms making it difficult for people to feel integrated into society and/or feel overly regulated, with four typologies of suicide along the axes of integration and regulation: altruistic, egoistic, fatalistic, and anomic suicide (56). Anthropological suicide research examines diverse meanings of suicide across cultures, in which the meaning of life and death are relational. Anthropologists also share the goal with sociologists to identify structural conditions and contexts that shape the intent, action and communal consequences of suicide.

Despite the vast types of suicide interventions, very few theories have informed the few existing Latinx-specific SP adult approaches, measures and interventions. Most of the SP interventions discussed here approached the Hispanic/Latinx population as a monolith and only two (35, 39) distinguished between nationalities (e.g., Puerto Ricans, Mexicans), geographies (U.S. Mexican-origin Latinx persons vs. Mexicans residing in Mexico), or immigrant status. We believe much of this is due to the lack of conceptual and theoretical application to their studies and interventions. We strongly encourage researchers to apply multidisciplinary theoretical and conceptual frameworks about suicide, but also those that theorize about the complex diversity of U.S. Latinx population such experiences of immigration status, race, racism and colorism, and sexuality (44, 57–59). This will not only encourage the creation of holistic suicide prevention but take into considerations interventions that address the barriers that multiply marginalized Latinx persons occupy, such as the exclusions tied to being unauthorized immigrants, formerly incarcerated persons, and active-duty military or veterans.

To prevent suicide in U.S. Latinx populations, we need more interventions that specifically address the sources of suicide for Latino/x populations and that are aligned with their cultural values and beliefs around mental health and wellbeing. However, this could be challenging given the age, cultural (different racial groups, nationalities, language), immigration status, and socioeconomic (occupation, educational status) positions, in the US Latinx population and geographic differences in social welfare and healthcare programs between U.S. states. Since suicide prevention needs to facilitate integration and reduce overregulation, we need interventions that address the different forms of structural and interpersonal discrimination that Latinx adults face in the United States, too (60).

Of the evidence-based interventions that are listed in the *CDC Suicide Prevention Resource for Action*, the evaluation of the school-based intervention, Signs of Suicide, did not show improvement in the Hispanic/Latinx students. Evidence-based interventions from the *CDC Suicide Prevention Resource for Action* may be universally applied to the U.S. population, but we need to discover why their approaches are not reducing Latinx adults’ sources of suicidal ideation, decreasing mental health stigma, or increasing Latinx adults’ help-seeking. Although it is best to create multiple adaptations of interventions with community participation to meet the needs of a diverse racial-ethnic group like the Hispanic/Latinx population, this may not be financially and logistically feasible. Nonetheless, given the size and growth of the Latinx population in in the United States, more existing evidence-based SP approaches, measures and interventions need to be culturally modified for the Latinx population, beyond language translation.

Cultural adaptation or modification, unlike culturally specific interventions, modify an original intervention to incorporate important aspects of a culture to be congruent with that group’s cognitive schemas, beliefs, norms and meanings about suicide and mental health, as well as those of ideal mental health (61). Barrera and colleagues (61) outline the steps to conduct and evaluate cultural adaptations for behavioral health: information gathering, preliminary adaptation design, preliminary adaptation tests, adaptation refinement, and cultural adaptation trial. Most cultural adaptations in this review focused on translating a measure or contents of an intervention into Spanish. None of the articles reviewed here discussed how best practices, like that of Barrera and colleagues, to modify an intervention, measure, or approach. Language translation alone will not capture lay health beliefs and practices around mental health and U.S. Latinx sources of resilience such as family, work ethic, and expressive culture.

The limitations of our review include that we had a change in authors. Graduate students transitioned into other employment and educational opportunities and there was a transition in the literature review team between 2023 and 2024. Nonetheless, our interrater reliability test indicates that there was a high congruence between coders in the selection of articles for this review. There was also a constant inclusion of the previous students in the production of the manuscript. Another limitation is that we focused exclusively on the U.S.-based Latinx population. In our initial sample of over 4,739 articles most of the SP approaches and interventions were focused on Latina/x youth, which is important given the suicide disparities between Latina/x youth and other racial-ethnic groups in the United States. We also need to start intervention early in life. It is possible that there were other SP measures and systematic reviews have been completed reviewing the literature on Hispanic/Latinx youth in the United States and abroad. We also did not include SP approaches and interventions used in other countries. There may be suicide prevention approaches and interventions in other Spanish-speaking Latin American countries that could be applicable to the U.S. population. However, the United States mental healthcare system is so distinct from those in Latin America that they may not be applicable to those Latinx born and residing in the United States.
